# Euthanasia

**Published:** 1874-09

**Authors:** 


Euthanasia.—* * * We write this, and at this time, because there has recently appeared in England an essay, entitled "Euthanasia," written by Mr. S. I. Williams, Jr., and published under the auspices of a Mrs. Crayshaw, who is said to be the wife of one of the millionaire iron and coal owners of Wales. In this publication the right, or rather duty, of suicide is set forth, the claim being "based on the paramount duty of doing all that is in our power to lessen the amount of physical suffering in the world." * * *

"That in all cases of hopeless and painful illness, it should be the recognized duty of the medical attendant, whenever so desired by the patient, to administer chloroform, or such other anæsthetic as may by-and-by supersede chloroform, so as to destroy consciousness at once, and put the sufferer to a quick and painless death; all needful precautions being adopted to prevent any possible abuse of such duty, and means being taken to establish, beyond the possibility of doubt or question, that the remedy was applied at the express wish of the patient."—*Philadelphia Medical Times—June.*

